# Diabetic Peripheral Neuropathy: Mechanisms and Emerging Therapies

**DOI:** 10.3390/biology15090723

**Published:** 2026-05-02

**Authors:** Mohammed M. H. Albariqi, Ibrahim A. Alradwan, Saad M. Alqahtani, Majed A. Majrashi, Basem Jahz Almutiri, Amjad Jabaan, Sultan Alzahrani

**Affiliations:** 1 Applied Genomics Technologies Institute, Health Sector, King Abdulaziz City for Science and Technology (KACST), Riyadh 11442, Saudi Arabia; balmutiri@kacst.gov.sa (B.J.A.);; 2 Digital Health Institute, Health Sector, King Abdulaziz City for Science and Technology (KACST), Riyadh 11442, Saudi Arabiaszahrani@kacst.gov.sa (S.A.); 3 Advanced Diagnostics and Therapeutics Technologies Institute, Health Sector, King Abdulaziz City for Science and Technology (KACST), Riyadh 11442, Saudi Arabia; ialradwan@kacst.gov.sa; 4 Bioengineering Institute, Health Sector, King Abdulaziz City for Science and Technology (KACST), Riyadh 11442, Saudi Arabia

**Keywords:** diabetic peripheral neuropathy, type 2 diabetes mellitus, hyperglycemia, dyslipidemia, microvascular, amyloidogenic, human islet amyloid polypeptide, antioxidants, anti-inflammatory agents

## Abstract

Diabetic peripheral neuropathy (DPN) is a common and debilitating complication of diabetes mellitus which affects individuals with both type 1 and type 2 diabetes mellitus (T2DM), presenting with sensory loss, pain, and progressive nerve dysfunction. DPN pathogenesis is multifactorial: chronic hyperglycemia activates the polyol, hexosamine, and protein kinase C (PKC) pathways, increases advanced glycation end-products, and drives oxidative stress, mitochondrial dysfunction, inflammation, and impaired neurotrophic signaling. In addition to hyperglycemia-driven mechanisms, dyslipidemia and microvascular insufficiency exacerbate neural ischemia and metabolic stress. Recent mechanistic, animal, and associative human studies further implicate amyloidogenic toxicity, particularly from human islet amyloid polypeptide (hIAPP), as a plausible contributory factor in peripheral nerve degeneration in T2DM, linking protein misfolding and aggregation to axonal damage and demyelination in DPN. Despite increased understanding of these mechanisms, current treatments remain mainly symptomatic. Emerging therapeutic strategies, including antioxidants, anti-inflammatory agents, modulators of mitochondrial function, amyloid oligomer modulators, neurotrophic enhancers, and regenerative approaches such as stem cells and gene-based therapies, offer potential to modify disease progression. The strength of evidence across these methods varies, ranging from mechanistic and animal studies to early human research and, in some cases, randomized clinical trials. Therefore, although several candidates show potential to alter the disease, few have demonstrated consistent benefits on objective measures of nerve structure or function in large clinical trials. This review summarizes the key mechanisms driving DPN in T2DM and highlights promising therapeutic innovations poised for clinical translation.

## Simple Summary

Diabetic peripheral neuropathy (DPN) is one of the most common complications of diabetes and can cause pain, numbness, and progressive nerve damage, significantly affecting patients’ quality of life. The condition develops through multiple biological processes triggered by long-term high blood sugar, including metabolic disturbances, oxidative stress, inflammation, and reduced blood supply to nerves. In people with type 2 diabetes, abnormal protein aggregation from human islet amyloid polypeptide (hIAPP) may also contribute to nerve injury. Despite advances in understanding these mechanisms, most current treatments focus only on managing symptoms rather than stopping disease progression. Researchers are now exploring new therapeutic strategies, such as antioxidants, anti-inflammatory agents, mitochondrial modulators, amyloid oligomer modulators, and regenerative approaches, including stem cell and gene-based therapies. These emerging approaches aim to better protect nerves and potentially modify the course of the disease. By summarizing current knowledge and highlighting promising treatments, this review helps guide future research toward more effective therapies for DPN.

## 1. Introduction

Diabetic peripheral neuropathy (DPN) is a common, progressive, and disabling complication that can develop over the lifetime of individuals with both type 1 and type 2 diabetes mellitus, characterized by involvement of the somatosensory nervous system and a marked reduction in quality of life [1,2,3]. It typically manifests as a length-dependent, symmetrical neuropathy with a stocking–glove distribution, in which distal lower limbs are affected first due to the particular vulnerability of long axons [4,5]. Patients frequently experience a combination of positive and negative sensory symptoms, including burning or electric shock-like pain, allodynia, hyperalgesia, paresthesia, numbness, and loss of protective sensation. These patterns reflect heterogeneous involvement of small and large fibers and contribute to gait disturbance, sleep disruption, mood disorders, foot ulceration, and increased risk of lower-limb amputation [1,5].

The pathogenesis of DPN is multifactorial and remains incompletely understood [5]. Chronic hyperglycemia is a central driver, activating interconnected metabolic pathways that promote oxidative stress, mitochondrial dysfunction, inflammation, and neurovascular injury. Additional factors, including dyslipidemia, obesity, impaired insulin signaling, and microvascular insufficiency, amplify these pathogenic processes and accelerate nerve damage [1,5]. These metabolic and vascular insults disrupt axonal transport, myelin integrity, and neurotrophic support, leading to progressive axonal degeneration and neuronal loss [1,4,5]. The function of hIAPP in DPN has still not been fully established. Unlike the well-supported roles of hyperglycemia-driven metabolic injury, oxidative stress, dyslipidemia, and microvascular dysfunction, current evidence for hIAPP remains limited and is based mainly on recent mechanistic, animal, and associative human studies [6]. Thus, hIAPP is best considered an emerging contributor, particularly in T2DM, that still requires further experimental and clinical validation [7,8].

Current management of DPN remains largely symptomatic and focuses on improving the metabolic milieu, alleviating neuropathic pain, and preventing foot complications [9]. Pharmacological treatment of painful DPN centers on agents that modulate nociceptive processing, including gabapentinoids (gabapentin and pregabalin), serotonin–noradrenaline reuptake inhibitors, tricyclic antidepressants, and certain sodium-channel-targeting drugs, complemented in selected cases by topical agents such as capsaicin or lidocaine [10]. Non-pharmacological interventions, such as structured exercise programs, weight management, neuromodulation techniques, and multidisciplinary foot-care strategies, provide important adjunctive benefits for symptom control and complication prevention [11].

Beyond symptomatic relief, increasing attention is being given to disease-modifying approaches that directly target key pathogenic pathways [12]. Investigational strategies include antioxidants and free-radical scavengers, agents that enhance mitochondrial function and bioenergetics, modulators of polyol and AGE-RAGE signaling, amyloid oligomer modulators, and neurotrophic or neuroprotective compounds that aim to preserve or restore neuronal and Schwann cell integrity [13,14]. Regenerative and reparative approaches, encompassing stem-cell-based therapies, gene therapy, and interventions designed to promote axonal regeneration and remyelination, are also under active exploration [15,16].

In this review, we examine the complex pathogenesis of DPN and the limitations of current clinical management. By exploring recent advances in understanding metabolic, vascular, and amyloidogenic mechanisms, as well as emerging therapeutic strategies, including antioxidants, neurotrophic agents, mitochondrial modulators, and regenerative approaches, we aim to highlight potential avenues for disease-modifying interventions and improved patient outcomes.

For this narrative review, relevant literature was identified through targeted searches of PubMed/MEDLINE, Scopus, Web of Science, and Google Scholar from database inception to February 2026. Search terms included combinations of “diabetic peripheral neuropathy,” “type 2 diabetes,” “hyperglycemia,” “dyslipidemia,” “oxidative stress,” “mitochondrial dysfunction,” “neuroinflammation,” “microvascular dysfunction,” “human islet amyloid polypeptide”, “hIAPP,” “amyloid,” “neurotrophic therapy,” “stem cell therapy,” “gene therapy,” “GLP-1 receptor agonist,” “SGLT2 inhibitor,” and “dis-ease-modifying therapy.” Priority was given to peer-reviewed mechanistic studies, animal models, clinical trials, systematic reviews, meta-analyses, clinical guidelines, and high-quality narrative reviews relevant to DPN pathogenesis, diagnosis, phenotyping, and emerging therapies. Additional references were identified from the bibliographies of selected articles. Because the objective was to provide a mechanistic and translational synthesis rather than a formal systematic review, no meta-analysis or structured risk-of-bias assessment was performed.

## 2. Clinical Features and Classification of Diabetic Peripheral Neuropathy

DPN is one of the most frequent long-term complications of diabetes mellitus, particularly in individuals with T2DM, and is a major cause of neuropathic pain [1,17]. The clinical progression of diabetic peripheral neuropathy (DPN) varies between type 1 and type 2 diabetes: in type 1, neuropathy generally arises following extended hyperglycemia and exhibits a more predictable response to rigorous glycemic control; conversely, in type 2, neuropathy may be present at diagnosis, frequently advances despite glucose optimization, and is exacerbated by concurrent metabolic risk factors such as dyslipidemia, obesity, and insulin resistance [18,19,20,21]. Neuropathic pain arises from lesions or diseases affecting the somatosensory system, without the presence of noxious stimuli, and results from the aberrant activation of peripheral nerves and the dorsal root pathway. It can be broadly categorized into peripheral neuropathic pain, involving damage to sensory nerves from the spinal cord to distal organs, and central neuropathic pain, which arises from lesions within the spinal cord or brain [22].

DPN is associated with a broad range of sensory disturbances. These include allodynia (pain from normally non-painful stimuli), hyperalgesia (exaggerated pain response to painful stimuli), paresthesia (abnormal sensations such as tingling or “pins and needles”), and hypoesthesia (reduced sensory perception). These symptoms may occur simultaneously due to damage to different classes of sensory neurons [23]. For example, patients may experience both allodynia and numbness in the same region, reflecting the complex relationship between nerve injury and impaired sensory transmission. Such abnormalities significantly impact daily functioning and increase the risk of unrecognized injuries due to diminished sensory awareness.

Diabetic neuropathies are heterogeneous in presentation, pathophysiology, and anatomical involvement. Thomas and Boulton proposed a widely used classification that divides diabetic neuropathy into generalized, focal, and multifocal forms [24,25]. Generalized neuropathy includes the classic DPN phenotype, characterized by symmetrical, length-dependent sensory loss affecting distal extremities. It also includes autonomic neuropathy, which involves dysfunction of involuntary physiological processes such as cardiovascular regulation, gastrointestinal motility, and bladder control [24,25]. Focal and multifocal neuropathies involve isolated or multiple peripheral nerves and may include nerve roots, resulting in mononeuropathies, radiculopathies, or polyradiculopathies [24,25,26,27].

The clinical manifestations of DPN depend on the specific peripheral nerve fibers affected, including sensory, motor, and autonomic. Sensory symptoms commonly include allodynia, paresthesia, hyperalgesia, and spontaneous pain. These symptoms often arise due to peripheral nerve degeneration, central sensitization, inflammation, altered neurotransmission, and microvascular dysfunction of the vasa nervorum [23,28]. In addition to painful symptoms, patients may experience reduced sensitivity, weakness, or complete loss of sensation as neural signal transmission becomes impaired due to progressive damage to nerve fibers. The coexistence of both heightened pain responses and diminished sensory responses reflects the complex and heterogeneous neurophysiology of DPN [29].

Diagnosis of DPN relies on characteristic symptoms, neurological examination, and objective testing. Nerve conduction studies (NCSs) remain the gold standard for identifying large-fiber neuropathy [30]. Additional assessments, including vibration perception thresholds, the 10-g monofilament test, and clinical evaluation of sensory modalities such as touch, pressure, temperature, pain, and vibration, complement NCSs and enable a more comprehensive characterization of fiber involvement [31]. For small-fiber neuropathy, quantification of intraepidermal nerve fiber (IENF) density in skin biopsies is a validated and widely adopted diagnostic tool, enabling early detection of small-fiber degeneration before abnormalities appear in NCSs [19]. Beyond conventional neurologic and electrophysiologic testing, attention is increasingly turning to simple blood-based indices that may support DPN detection and risk stratification. Among these, the C-reactive protein-to-albumin ratio (CAR) and prognostic nutritional index (PNI) have shown the clearest diagnostic signals in recent studies [32]. In type 2 diabetes cohorts, a CAR test identified diabetic neuropathy with 78% sensitivity and 73% specificity, while a PNI showed 81% sensitivity and 84% specificity [32,33]. Lower PNI levels have also been associated with increased DPN risk and poorer nerve conduction parameters [34]. The controlling nutritional status (CONUT) score and the uric acid-to-HDL cholesterol ratio (UHR) are likewise emerging as composite indicators of nutritional-inflammatory and metabolic dysfunction, and a higher UHR has been associated with diabetic neuropathy and abnormalities in nerve conduction studies [33,34]. However, compared with CAR and PNI, the current evidence for CONUT and UHR in DPN remains less standardized, and these indices should presently be regarded as complementary and investigational biomarkers rather than substitutes for established diagnostic methods such as clinical examination, nerve conduction studies, or small-fiber testing [33]. Together, these clinical features and diagnostic approaches provide the foundation for identifying DPN and distinguishing its heterogeneous presentations, enabling more precise assessment of disease severity and guiding appropriate management strategies.

## 3. Pathogenesis of Diabetic Peripheral Neuropathy

**Figure 1:**
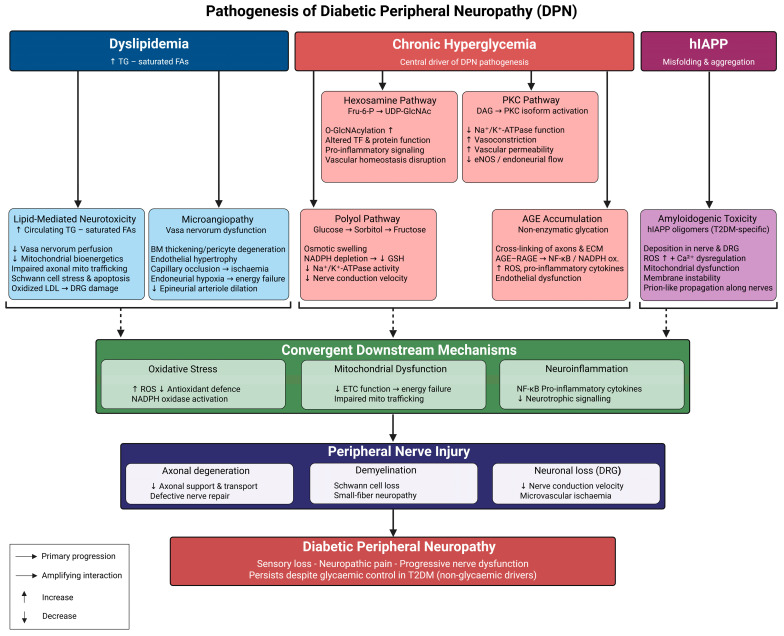
Pathogenesis of diabetic peripheral neuropathy (DPN). Chronic hyperglycemia, the central driver of DPN, activates interconnected metabolic pathways—including the polyol pathway, hexosamine biosynthetic pathway (HBP), protein kinase C (PKC), and advanced glycation end-product (AGE) formation—thereby increasing reactive oxygen species (ROS), mitochondrial dysfunction, endothelial impairment, and nuclear factor-κB (NF-κB)-mediated inflammation. Non-glycemic contributors—including dyslipidemia, microvascular injury of the vasa nervorum, and amyloidogenic toxicity from human islet amyloid polypeptide (hIAPP)—further exacerbate oxidative stress and neuroinflammation. These convergent mechanisms culminate in axonal degeneration, demyelination, dorsal root ganglion (DRG) neuronal loss, and progressive peripheral nerve dysfunction characteristic of DPN. Abbreviations: DPN, diabetic peripheral neuropathy; HBP, hexosamine biosynthetic pathway; PKC, protein kinase C; AGE, advanced glycation end-product; ROS, reactive oxygen species; NF-κB, nuclear factor-κB; hIAPP, human islet amyloid polypeptide; DRG, dorsal root ganglion; TGs, triglycerides; FAs, fatty acids; ETC, electron transport chain; NADPH, nicotinamide adenine dinucleotide phosphate; GSH, glutathione; eNOS, endothelial nitric oxide synthase.

Diabetic peripheral neuropathy (DPN) is comprehensively elucidated through a three-tier mechanistic paradigm, progressing from initial metabolic insults, through interrelated biochemical amplifiers, to convergent effectors of structural nerve damage [5,13,26] (Table 1). At the primary level, chronic hyperglycemia and dyslipidemia represent essential upstream conditions that must be present for the downstream pathological cascade to occur: sustained hyperglycemia facilitates excessive glucose flow into pathological biochemical pathways [21,35], while dyslipidemia, through free fatty acid-mediated lipotoxicity and disrupted sphingolipid metabolism, independently undermines neuronal and Schwann cell homeostasis [36]. The initial shocks subsequently trigger a network of interrelated, mutually reinforcing biochemical amplifiers that characterize the second layer. Increased intracellular glucose swiftly activates the polyol pathway, leading to sorbitol buildup, osmotic stress, and redox imbalance [37,38,39], while simultaneously stimulating protein kinase C (PKC) isoforms, which enhance oxidative signaling and compromise vascular function [40] (Table 1). The hexosamine pathway and the synthesis of advanced glycation end-products (AGEs), along with the subsequent activation of its receptor RAGE, function as enduring amplifiers, maintaining oxidative stress and inflammatory signaling across prolonged periods of chronic exposure [41,42]. Mitochondrial dysfunction and neuroinflammation act as downstream convergent amplifiers, perpetuating themselves through feed-forward mechanisms involving the overproduction of reactive oxygen species (ROS) and NF-κB-mediated cytokine release [43,44,45], while microvascular injury connects metabolic and vascular processes, exacerbating neuronal energy deficits [46]. Emerging evidence further indicates that the amyloidogenic processing of human islet amyloid polypeptide (hIAPP) may serve as a non-glycemic amplifier functioning concurrently; however, this mechanism is still speculative rather than a confirmed pathway [7,8] (Table 1). The cumulative effect of these heightened signals results in structural nerve damage at the tertiary level: axonal degeneration, demyelination, Schwann cell depletion, dorsal root ganglion neuronal loss, and diminished axonal regenerative ability collectively manifest the clinical phenotype of diabetic peripheral neuropathy (DPN) (Figure 1) [13,14,28].

Operationally, this framework is intended to connect dominant mechanisms with clinically relevant phenotypes and therapeutic decision points. Early hyperglycemia-driven polyol and PKC activation is most relevant to small-fiber-predominant and painful presentations, whereas sustained AGE–RAGE and hexosamine signaling may be more prominent in long-duration, painless, progressive sensory loss and ulcer-risk phenotypes. Mitochondrial dysfunction is most closely aligned with progressive axonal, length-dependent neuropathy, while neuroinflammation is particularly relevant to painful DPN with nociceptor sensitization. hIAPP-related amyloidogenic toxicity should be regarded as a hypothesis-generating, T2DM-enriched mechanism until DPN-specific validation is available. Thus, the three-tier model should guide patient stratification, the selection of mechanism-matched therapeutic strategies, and the choice of trial endpoints, rather than serving only as a descriptive schema (Table 1).

### 3.1. Hyperglycemia-Induced Metabolic Derangements (An Interlinked Metabolic Network)

Chronic hyperglycemia is a central driver of the metabolic disturbances that culminate in DPN. Sustained elevation of blood glucose disrupts normal cellular metabolism and activates several maladaptive biochemical pathways. Among the biochemical pathways activated by hyperglycemia, the polyol pathway and protein kinase C (PKC), particularly the beta isoform, are considered primary effectors: both are activated within minutes to hours of intracellular glucose elevation, both act directly on neural and endoneurial vascular targets, and both have been specifically targeted in prospective interventional trials [37,38,39]. On the other hand, the hexosamine pathway and the AGE–RAGE axis are best understood as secondary and temporally sustained amplifiers: they require prolonged hyperglycemic exposure to exert their full pathogenic effects and contribute substantially to the perpetuation rather than the initiation of nerve injury [41,42]. Collectively, these promote convergent downstream amplifiers: oxidative stress, mitochondrial dysfunction, inflammatory signaling, and vascular abnormalities that contribute to the development of DPN [13,35]. The following sections will discuss these pathogenic pathways.

#### 3.1.1. Polyol Pathway

In the polyol pathway, excess intracellular glucose is reduced to sorbitol, which is subsequently oxidized to fructose. Overactivation of this pathway in diabetes leads to intracellular sorbitol accumulation, which exerts osmotic effects that cause cellular swelling and structural damage in peripheral nerves. In parallel, increased flux through the polyol pathway consumes NADPH, a critical cofactor required for antioxidant systems, including glutathione reductase. Depletion of NADPH weakens endogenous antioxidant defenses, thereby amplifying oxidative stress. Accumulation of sorbitol and fructose also disrupts the activity of Na^+^/K^+^-ATPase, impairing maintenance of the axonal transmembrane electrochemical gradient and contributing to reduced nerve conduction velocity in DPN [13,37,38] (Table 1).

#### 3.1.2. Hexosamine Pathway

Hyperglycemia additionally diverts fructose-6-phosphate into the hexosamine pathway, where it is converted to uridine diphosphate N-acetylglucosamine (UDP-GlcNAc). Elevated UDP-GlcNAc enhances O-GlcNAcylation of nuclear and cytoplasmic proteins, including key transcription factors and signaling molecules. This post-translational modification alters gene expression and protein function, thereby modulating pathways involved in inflammation, oxidative stress responses, and vascular homeostasis. Dysregulated O-GlcNAc signaling in neural and vascular cells of the peripheral nervous system is therefore implicated in the chronic inflammatory and vasculopathic milieu that contributes to nerve injury in DPN [13,39].

#### 3.1.3. Protein Kinase C (PKC) Pathway

Concurrently, increased glucose metabolism elevates glyceraldehyde-3-phosphate levels, promoting diacylglycerol (DAG) synthesis and sustaining PKC isoform activation. PKC overactivation impairs Na^+^/K^+^-ATPase function and disrupts neurovascular regulation by inducing vasoconstriction, increasing vascular permeability, and altering endothelial nitric oxide bioavailability. These changes compromise endoneurial blood flow and aggravate ischemic injury to peripheral nerves [13,40] (Table 1).

#### 3.1.4. Advanced Glycation End-Products and Downstream Effects

In parallel with polyol, hexosamine, and PKC pathways, chronic hyperglycemia accelerates non-enzymatic glycation of proteins, lipids, and nucleic acids, leading to the formation and accumulation of AGEs. Within the peripheral nervous system, AGEs localize to axons, Schwann cells, the neural microvasculature, and extracellular matrix components. Covalent modification of these structures induces irreversible cross-linking, increased stiffness, and reduced elasticity of nerve and vascular tissues, thereby impairing axonal support, nerve fiber regeneration, and microvascular integrity [13,41,42] (Table 1).

Beyond direct structural damage, AGEs exert potent signaling effects through interaction with their principal receptor, RAGE. AGE–RAGE engagement activates downstream pathways that include NF-κB signaling and NADPH oxidase, promoting reactive oxygen species production, upregulation of pro-inflammatory cytokines, and endothelial dysfunction. This pro-oxidant, pro-inflammatory state exacerbates microvascular injury, reduces nutritive blood flow to peripheral nerves, and further compromises nerve conduction. Thus, AGE accumulation and AGE–RAGE signaling represent a critical axis by which hyperglycemia amplifies oxidative stress, inflammation, and vascular dysfunction, driving the progression of DPN [13,42].

### 3.2. Role of Dyslipidemia and Microvascular Insufficiency

#### 3.2.1. Lipid-Mediated Neurotoxicity

Dyslipidemia is increasingly recognized as an important contributor to the development and progression of DPN. Elevated serum triglycerides—observed even in non-obese patients with DPN—are associated with reduced myelinated fiber density and worsening nerve structural integrity. In both clinical and experimental models, high levels of circulating saturated and unsaturated fatty acids promote endothelial dysfunction and impaired microvascular perfusion, which likely compromises blood flow within the vasa nervorum, particularly those supplying the sural nerve [13,29,36].

Beyond their vascular effects, excess fatty acids exert direct neurotoxicity through multiple mechanisms. In sensory neurons, elevated lipid concentrations disrupt mitochondrial bioenergetics and impair mitochondrial trafficking from the cell body to distal axons, thereby compromising energy availability at nerve terminals. Schwann cells exposed to long-chain fatty acids similarly develop mitochondrial dysfunction, endoplasmic reticulum stress, and oxidative injury, ultimately leading to apoptosis. Additionally, oxidized lipoproteins induce further oxidative damage within dorsal root ganglion neurons, affecting proteins, nucleic acids, and lipid membranes. These combined effects contribute to axonal degeneration, small-fiber neuropathy, and progressive neural dysfunction [13,29,36].

#### 3.2.2. Microangiopathy and Nerve Ischemia

Microvascular insufficiency is another key pathogenic driver of DPN. Diabetes-related microangiopathy affects the vasa nervorum, the specialized microvessels that supply peripheral nerves, leading to chronic nerve ischemia and hypoxia. Structural abnormalities observed in sural nerve biopsies of individuals with diabetes include thickening of the endoneurial capillary basement membrane, endothelial hypertrophy and hyperplasia, pericyte degeneration, and capillary occlusion. These vascular changes impair nutrient and oxygen delivery, resulting in metabolic stress within peripheral nerves [13,46].

Endoneurial hypoxia has been directly linked to reduced glucose and oxygen uptake by neural cells, contributing to energy failure and axonal injury. Interestingly, increased endoneurial capillary density has been observed in diabetic patients, suggesting a compensatory response to ischemic stress; however, this adaptive mechanism appears insufficient to normalize perfusion. Experimental studies further indicate that impaired vasodilation of epineurial arterioles precedes measurable decreases in nerve conduction velocity, supporting the notion that microvascular dysfunction is an early and pivotal event in DPN pathogenesis [13,46] (Table 1).

Together, dyslipidemia-induced neurotoxicity and diabetes-associated microvascular abnormalities underscore the intertwined metabolic and vascular contributions to nerve injury in DPN [13,46].

### 3.3. Amyloidogenic Mechanisms in DPN

Growing evidence suggests that DPN in T2DM does not be fully explained by hyperglycemia alone, although the strength of evidence varies among non-glycemic mechanisms. Unlike type 1 diabetes, where intensive glycemic control significantly reduces neuropathy risk. DPN in T2DM often persists or progresses despite improved glycemic control. Additionally, neuropathic changes have been reported in individuals with prediabetes, further indicating the involvement of non-glycemic mechanisms. One emerging contributor is amyloidogenic toxicity, particularly from human islet amyloid polypeptide (hIAPP). T2DM is increasingly recognized not only as a metabolic disorder but also as an amyloid-associated disease, as hIAPP aggregates accumulate in pancreatic islets [8]. Recent studies indicate that misfolded hIAPP may exert systemic effects, extending beyond pancreatic β-cell destruction [7].

At the mechanistic level, oligomeric hIAPP species share structural and toxic properties with other amyloidogenic proteins such as amyloid-β and α-synuclein, both well-established mediators of neurodegeneration [8]. Experimental data suggest that circulating hIAPP can deposit in peripheral nerves and dorsal root ganglia, where it promotes oxidative stress, mitochondrial dysfunction, calcium homeostasis disruption, and membrane instability [8]. These processes might compromise neuronal integrity and Schwann cell function, contributing to demyelination, impaired axonal support, and defective nerve repair. In parallel, amyloid-induced microvascular injury may aggravate endoneurial ischemia, thereby amplifying nerve damage [7,8] (Table 1).

The concept that amyloid deposition drives neuropathy is supported by longstanding observations in familial and acquired amyloid polyneuropathies, providing a mechanistic precedent for similar processes in diabetes [8]. Emerging human and animal studies in T2DM report associations between elevated circulating hIAPP levels and increased severity of DPN. Furthermore, amyloidogenic stress can induce sensory neuron loss partly independent of glycemic status. Co-aggregation of hIAPP with neuronal proteins has also been proposed to facilitate prion-like propagation of toxic species along peripheral nerves, potentially explaining the progressive and spreading nature of neuropathic changes [7,8].

The neurotoxicity evidence implicating hIAPP in peripheral nerve injury derives predominantly from in vitro cell culture systems and transgenic animal models engineered to overexpress human IAPP [7,8]; however, these experimental conditions may not faithfully recapitulate the spatiotemporal dynamics of hIAPP aggregation, fibril exposure geometry, or tissue distribution encountered in human peripheral nerve in vivo, and transgenic overexpression inherently produces supraphysiological hIAPP concentrations that may not reflect the pathophysiologically relevant range present in type 2 diabetes. Beyond these model-level constraints, the magnitude of hIAPP’s contribution to DPN in clinical populations has not been established through large-scale prospective human cohort studies incorporating direct neural tissue sampling or validated neurological endpoints such as intraepidermal nerve fiber density; correlative evidence linking circulating IAPP levels or pancreatic amyloid burden to DPN severity remains limited and has not been consistently replicated across independent cohorts. Furthermore, no clinical trial has yet evaluated whether hIAPP aggregation inhibition or anti-amyloid intervention confers measurable benefit on DPN outcomes in human subjects, leaving the entire translational pathway from mechanistic hypothesis to clinical efficacy untested at the trial level. Accordingly, hIAPP-related mechanisms should be understood as a hypothesis-generating, non-glycemic extension of the DPN pathogenic framework, one that expands the conceptual scope of the disease beyond purely glycemic pathways [7,8], but cannot currently be considered an established equivalent of the polyol, PKC, AGE–RAGE, or mitochondrial dysfunction pathways; prospective cohort studies with intraepidermal nerve fiber density endpoints, systematic measurement of circulating IAPP levels in patients with DPN, and ultimately interventional trials targeting hIAPP aggregation all warrant pursuit before any stronger causal claim may be advanced (Table 1).

### 3.4. Convergent Downstream Mechanisms

#### 3.4.1. Oxidative Stress

The state of chronic hyperglycemia induces excessive generation of ROS via the uncoupling process of the ETC, where electrons produced by Complex I and Complex III react directly with oxygen molecules to generate superoxide anions (O_2_•−) without participating in oxidative phosphorylation [39,47]. Simultaneously, the acceleration of the polyol pathway by aldose reductase leads to a massive consumption of NADPH. As the coenzyme necessary for regenerating reduced glutathione (GSH) from oxidized glutathione (GSSG) is consumed, GSH levels drop, rendering the reduction of hydrogen peroxide (H_2_O_2_) and lipid peroxides difficult, thus exacerbating oxidative damage to the peripheral nerve cell membranes and proteins [39]. DNA single-strand breaks stimulate poly (ADP-ribose) polymerase (PARP), consuming NAD^+^ in an attempt to repair the damaged DNA strands, eventually leading to exhaustion of both NAD^+^ and ATP pools. Lack of NAD^+^ prevents the function of glyceraldehyde-3-phosphate dehydrogenase (GAPDH), which in turn redirects glycolysis to other ROS production pathways. Superoxide interacts with nitric oxide (NO) to produce peroxynitrite (ONOO−), which nitrates tyrosine residues on proteins, initiates lipid peroxidation, and causes mitochondrial inner membrane damage [39,48,49]. Excessive ROS activity activates NF-κB, PKC, and AGE formation pathways, establishing crosstalk among them and multiplying injury. Peripheral neurons and Schwann cells are vulnerable due to their high energy consumption and low antioxidant capacity [39,47,50].

#### 3.4.2. Mitochondrial Dysfunction

The high concentration of glucose induces a direct effect on the structure and function of the mitochondrial respiratory chain, especially Complex I and Complex III, due to their sensitivity to oxidative modification by reactive oxygen species (ROS) [51]. In this case, the efficiency of electron transfer decreases, which results in the weakening of the proton gradient, followed by the suppression of oxidative phosphorylation and ATP synthesis in the DRG neurons and Schwann cells [52,53]. The process of mitochondrial fission begins in response to energy deficiency, which involves recruitment of dynamin-related protein 1 (DRP1) to the outer mitochondrial membrane. The number of fragments increases due to the lack of mitochondrial fusion through OPA1 and MFN2 [54]. Fragmented mitochondria demonstrate low mitochondrial membrane potential ΔΨm and calcium buffering capacity; also, the probability of outer membrane permeabilization is higher [54,55]. Mitochondrial quality control is disturbed: the inability of the PINK1/Parkin system to remove damaged organelles leads to their persistence, accumulation of free radicals, and additional oxidative stress in DRG neurons [52,53,54]. The loss of PGC-1α contributes to decreased biogenesis and mitochondrial content in DRG neurons. The deficiency in ATP synthesis impairs axonal transport, neurotrophic factor supply, and Na^+^/K^+^-ATPase activity [52,55] (see Table 1).

#### 3.4.3. Neuroinflammation

Chronic hyperglycemia creates a pro-inflammatory milieu in peripheral nerves via multiple converging processes [56]. The upregulation of monocyte chemoattractant protein-1 (MCP-1/CCL2) facilitates the infiltration of endoneurial macrophages, while resident macrophages polarize towards an M1 pro-inflammatory phenotype, marked by increased secretion of tumor necrosis factor-alpha (TNF-α), interleukin-1β (IL-1β), and interleukin-6 (IL-6) [57,58]. Nuclear factor-κB (NF-κB) functions as a pivotal transcriptional center triggered by reactive oxygen species (ROS), interaction of the AGE–RAGE receptor, and stimulation of Toll-like receptor 4 (TLR4) by damage-associated molecular patterns (DAMPs), including high-mobility group box protein 1 (HMGB1) and free fatty acids [56,59]. TLR4 is consistently expressed on Schwann cells and endoneurial macrophages, making these cells susceptible to metabolic danger signals commonly found in diabetic tissues [60,61]. The NLRP3 inflammasome is formed in macrophages and Schwann cells, facilitating the caspase-1-mediated cleavage of pro-IL-1β and pro-IL-18 into their active secretory forms, while downstream cyclooxygenase-2 (COX-2) enhances the inflammatory response [59]. TNF-α and IL-1β enhance peripheral nociceptor sensitivity by increasing TRPV1 and TRPA1 channel activity and diminishing inhibitory signaling, hence contributing to the unpleasant phenotype of diabetic peripheral neuropathy (DPN) [56,57]. Chronic neuroinflammation reduces Schwann cell synthesis of nerve growth factor (NGF) and brain-derived neurotrophic factor (BDNF), resulting in a milieu detrimental to axonal regeneration [58]. Anti-inflammatory approaches aimed at IL-1β and TLR4 exhibit preclinical effectiveness but lack validation in clinical studies for diabetic peripheral neuropathy (DPN) [60] (Table 1).

### 3.5. Peripheral Nerve Injury

#### 3.5.1. Axonal Degradation

Axonal degeneration in diabetic peripheral neuropathy exhibits a characteristic length-dependent, dying-back pattern, where structural and functional failure commences at the most distant nerve terminals and progresses retrogradely towards the neuronal cell body [62]. Hyperglycemia and increased reactive oxygen species damage the microtubular cytoskeleton in axons, hindering kinesin-mediated anterograde transport of mitochondria, synaptic vesicles, and neurotrophic factors, as well as dynein-mediated retrograde transport of signaling payloads [63]. The failure of anterograde transport deprives distal axonal compartments of energy sources and survival signals, hastening terminal degeneration [62,63]. One of the initial measurable structural alterations is a decrease in intraepidermal nerve fiber density (IENFD), evaluated using skin punch biopsy with immunostaining for protein gene product 9.5 (PGP9.5). IENFD loss effectively identifies small-fiber neuropathy prior to observable alterations in nerve conduction velocity, serving as a sensitive biopsy-derived endpoint [62]. Injury to Schwann cells concurrently removes trophic support provided by nerve growth factor (NGF) and glial cell line-derived neurotrophic factor (GDNF), thus depriving axon terminals of essential signals for structural integrity [63]. Distal axonal degeneration initiates the SARM1 (sterile alpha and TIR motif-containing 1) axon destruction program, which facilitates NAD^+^ cleavage and propels the swift self-destruction of the severed distal segment via a Wallerian-like process [64,65,66]. The resulting pattern of sensory loss in the stocking-and-glove distribution indicates the selective susceptibility of the longest axons that innervate the feet and distal lower limbs [62].

#### 3.5.2. Demyelination

Schwann cells maintain the integrity of the myelin sheath by continuously synthesizing, compacting, and renewing lipid-rich membrane layers, operations that impose significant metabolic demands and render them susceptible to the biochemical disruptions associated with diabetic peripheral neuropathy [67,68]. Activation of protein kinase C (PKC) in Schwann cells phosphorylates and inhibits Na^+^/K^+^-ATPase, hence altering ion homeostasis, inducing cellular swelling, and disturbing the architecture of the myelin bilayer [69]. The accumulation of advanced glycation end-products (AGEs) cross-links myelin-associated proteins and disrupts the molecular signaling interface between Schwann cells and axons, partially sustained by myelin-associated glycoprotein (MAG), L1 cell adhesion molecule, and neural cell adhesion molecule (N-CAM) [67]. The disruption of this interface results in paranodal demyelination and the expansion of nodal gaps at the nodes of Ranvier [67]. Hyperglycemic circumstances inhibit the transcription of key structural myelin proteins, such as myelin basic protein (MBP), myelin protein zero (P0), and peripheral myelin protein 22 (PMP22), resulting in thinner and structurally impaired sheaths [70]. The electrophysiological correlate is a deceleration of saltatory conduction, measured as diminished nerve conduction velocity (NCV) in electrodiagnostic assessments. Remyelination after injury is further compromised due to diminished Schwann cell proliferation and dedifferentiation capacity, caused by oxidative damage and reduced neuregulin-1 (NRG1)/ErbB2 receptor signaling, which hinders myelin repair [71]. Large myelinated Aβ sensory fibers and motor axons are predominantly impacted, resulting in the deficiencies in vibration sensitivity and the decreases in nerve conduction velocity that typify severe illness [67,68].

#### 3.5.3. Neuronal Loss

Dorsal root ganglion (DRG) sensory neurons are structurally unprotected; in contrast to peripheral nerve trunks, they exist outside the blood-nerve barrier and maintain direct contact with the hyperglycemic bloodstream [72]. The exposure, along with the elevated metabolic activity and restricted regenerative capacity of post-mitotic neuronal cell bodies, makes DRG neurons especially vulnerable to apoptosis [72,73]. The intrinsic route is activated when oxidative stress and energy depletion induce the release of cytochrome c from the outer mitochondrial membrane, leading to apoptosome formation, activation of caspase-9, and subsequent cleavage of effector caspase-3, resulting in irreversible nuclear fragmentation [74,75]. A comparable mechanism occurs via endoplasmic reticulum (ER) stress: the buildup of misfolded proteins under hyperglycemic and oxidative circumstances activates the unfolded protein response (UPR) through its ATF6, IRE1α, and PERK pathways [74,75]. Inability to rectify proteostatic imbalance activates the transcription factor CHOP (C/EBP homologous protein), which facilitates the activation of apoptotic genes [74]. The diminished availability of nerve growth factor (NGF) and brain-derived neurotrophic factor (BDNF) reduces TrkA and TrkB receptor activation, hence weakening PI3K/Akt prosurvival signaling and decreasing the apoptotic threshold [72]. In contrast to peripheral axons, which possess a limited ability to regenerate, mature dorsal root ganglion neuronal cell bodies do not undergo division, resulting in a permanent decline in sensory function upon their loss [73]. The extent of DRG neuron loss is associated with the length of the disease and total glycemic exposure, aligning with the notion of metabolic memory [76].

### 3.6. Clinical Manifestations of Diabetic Peripheral Neuropathy

Diabetic peripheral neuropathy (DPN) presents through a wide spectrum of sensory, motor, and autonomic manifestations resulting from chronic hyperglycemia-induced nerve damage. The most prevalent form is distal symmetric polyneuropathy (DSPN), which affects up to 50% of diabetic patients and follows a characteristic “stocking-and-glove” distribution, beginning in the feet and ascending proximally [13]. Positive sensory symptoms include burning pain, paresthesia, allodynia, and hyperalgesia, while negative symptoms encompass numbness and progressive loss of protective sensation [77]. Neuropathic pain is typically bilateral, worsens nocturnally, and develops in approximately 20–40% of patients with documented neuropathy [28]. Motor involvement manifests as muscle weakness, reduced ankle reflexes, and gait disturbances, while autonomic dysfunction may cause orthostatic hypotension, gastroparesis, and urinary retention [78]. In severe cases, loss of protective sensation predisposes patients to foot ulceration, Charcot neuroarthropathy, and lower-limb amputation, underscoring the profound clinical burden of DPN [77].

### 3.7. Translational Implications of Pathogenic Mechanisms

The activation of the polyol and protein kinase C (PKC) pathways starts swiftly in response to an intracellular glucose surplus, mostly damaging small unmyelinated C fibers and thinly myelinated Aδ fibers before any significant impairment of large myelinated Aβ fibers is observed [20,23]. The clinical correlates are burning dysesthesia, thermal hypoesthesia, mechanical allodynia, and stocking-and-glove sensory loss within relatively preserved nerve conduction velocities [18,19,22]. Given that structural axonal damage is still minimal, therapies targeting polyols and PKC are most logically assessed in early-stage, small-fiber-predominant diabetic peripheral neuropathy, where recovery potential is maintained [21]. This window cannot be identified using nerve conduction investigations; however, intraepidermal nerve fiber density (IENFD) assessed via skin punch biopsy with PGP9.5 immunostaining and corneal confocal microscopy (CCM) can reveal small-fiber alterations prior to the onset of nerve conduction velocity (NCV) problems [79,80,81,82].

The accumulation of advanced glycation end-products (AGEs) and the activation of the receptor for AGEs (RAGE) occur gradually over years of hyperglycemic exposure, becoming particularly pronounced in individuals with prolonged disease duration and significant tissue damage [83]. This clinically corresponds to the painless diabetic peripheral neuropathy (DPN) phenotype, characterized by progressive distal sensory loss, proprioceptive deficits, autonomic dysfunction, and an increased risk of foot ulceration, all occurring without significant neuropathic pain—impacting around 50% of DPN patients who remain asymptomatic despite observable nerve fiber loss [17,18]. AGE–RAGE-targeted interventions are best suitable for individuals with chronic diabetes and a significant accumulation of tissue AGEs; skin autofluorescence provides a scalable non-invasive alternative, but its relationship with neurological outcomes necessitates prospective validation [83].

Mitochondrial failure in dorsal root ganglion neurons and Schwann cells—impeding ATP synthesis, disrupting axonal transport, and causing persistent ROS overproduction, propels the gradual, length-dependent, mainly axonal nerve degeneration characteristic of advanced diabetic peripheral neuropathy (DPN) [18,81]. Given that the principal pathological substrate is axonal survival rather than nociceptor sensitization, agents targeting mitochondria should be assessed based on structural endpoints: serial intraepidermal nerve fiber density (IENFD) and corneal confocal microscopy (CCM)-based sub-basal plexus quantification offer direct longitudinal measurements, while plasma neurofilament light chain (NfL) has been suggested as a pharmacodynamic biomarker for axonal injury, awaiting validation in diabetic peripheral neuropathy (DPN) trials [79,80,84].

Neuroinflammation, characterized by endoneurial macrophage infiltration, NF-κB-mediated cytokine release, and TRPV1/TRPA1-induced nociceptor sensitization, is the basis of the painful diabetic peripheral neuropathy (DPN) phenotype [22], which is clinically and physiologically distinct from painless DPN and necessitates phenotypically enriched populations. Anti-inflammatory therapies are best assessed in individuals with verified painful diabetic peripheral neuropathy and objective indications of endoneurial inflammation activity. The previously observed 30–50% placebo response rate in painful diabetic peripheral neuropathy trials can be mitigated through the enrichment of inflammatory biomarkers and the implementation of adaptive designs [19].

These tracks collectively reveal a singular structural deficiency: targets driven by mechanistic motivations have consistently been associated with nerve conduction velocity measurements endpoints that primarily reflect large-fiber function in established disease during a phase when the small-fiber pathology most pertinent to the targeted mechanism may no longer be the critical factor in observable decline. NCV lacks sensitivity to early DPN and does not effectively identify the patient profile most likely to exhibit a therapeutic response [82,85]. Rectifying this discrepancy necessitates a methodical alignment of mechanism-stratified patient selection, intervention timing prior to irreversible structural damage, and endpoint selection tailored to each pathogenic axis: IENFD and CCM for early small-fiber interventions; NfL for axonal injury; skin autofluorescence for AGE-axis trials; and pain NRS with biomarker-based enrichment for neuroinflammatory interventions [79,80,81,82,84,85].

Taken together, these observations suggest that DPN is unlikely to respond to a uniform therapeutic approach. Instead, dominant mechanisms may differ across patient subsets and may correspond to distinct clinical phenotypes. Early small-fiber-predominant disease may require interventions that target metabolic and mitochondrial stress before irreversible axonal loss; painless, long-duration neuropathy with tissue glycation burden may be more suitable for AGE–RAGE-directed strategies; painful inflammatory phenotypes may require anti-inflammatory or ion-channel-modulating approaches; and regenerative or gene-based therapies may be most relevant once structural nerve loss predominates. This phenotype-oriented approach provides a clinically actionable bridge between pathophysiology, trial design, and individualized therapeutic strategy.

**Table 1:** Mechanistic Pathways, Clinical Phenotypes, Therapeutic Targets, and Trial Endpoints in DPN.

Mechanism	Clinical Phenotype	Rational Therapeutic Target	Most Appropriate Trial Endpoint
Polyol/PKC	Early painful DPN, small-fiber loss	Aldose reductase inhibitors, PKC-beta inhibitors	IENFD, CCM, QST thermal thresholds
AGE–RAGE/Hexosamine	Insidious painless DPN, long duration	AGE-crosslink breakers, RAGE antagonists	Skin autofluorescence, NCV (large fiber), IENFD
Mitochondrial dysfunction	Progressive axonal DPN, length-dependent	MitoQ, elamipretide, benfotiamine, PGC-1α activators	Serial IENFD, CCM morphometry, NfL
Neuroinflammation	Painful DPN, autonomic involvement	IL-1β blockers, TLR4 antagonists, NLRP3 inhibitors	NRS pain score, PGIC, inflammatory biomarkers
Microvascular ischemia	Sensorimotor DPN, autonomic neuropathy	Vasodilators, VEGF gene therapy	MRN, nerve ultrasound, autonomic function tests
hIAPP (emerging)	Painful DPN (hypothesis)	IAPP aggregation inhibitors (preclinical)	IENFD, pain scores (no validated endpoint yet)

## 4. Current Clinical Management of DPN

Current therapeutic strategies for DPN are primarily directed at alleviating neuropathic pain and focus on treating disease symptoms to improve quality of life [18,86]. Most approved interventions target nociceptive signaling pathways downstream of axonal injury, Schwann cell dysfunction, and neurovascular compromise, without directly addressing the upstream metabolic, inflammatory, and microvascular drivers of nerve degeneration. Consistent with this limitation, even with optimized symptomatic treatment, progressive nerve fiber loss and sensory decline continue in patients, particularly those with long-standing type 2 diabetes or multiple cardiometabolic comorbidities [18].

**Figure 2:**
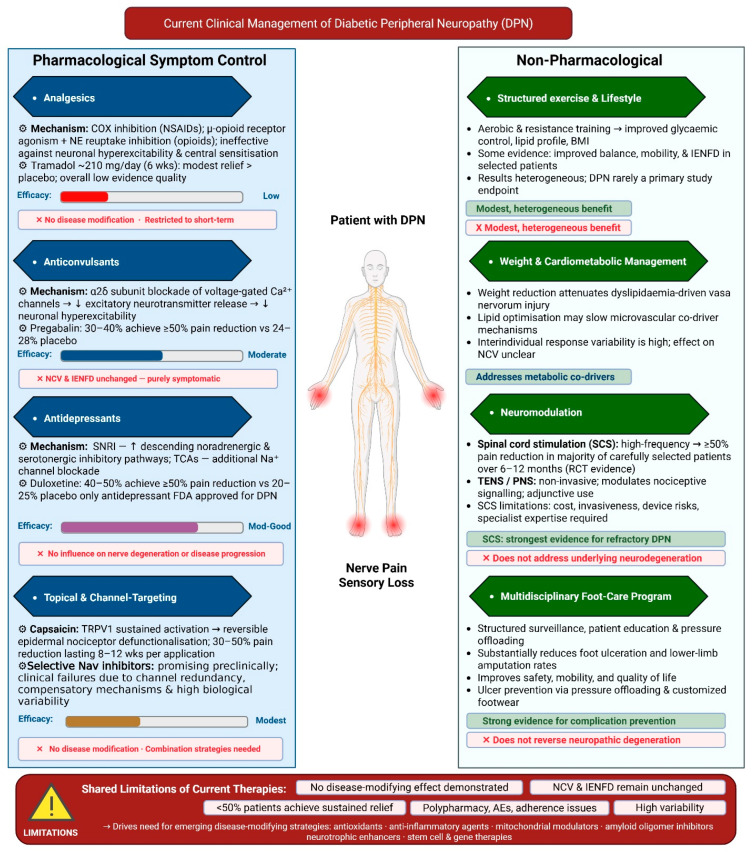
Current clinical management of diabetic peripheral neuropathy (DPN). DPN management is primarily multimodal and symptom-focused. Pharmacological therapies include analgesics, gabapentinoids, antidepressants such as duloxetine, and topical agents including capsaicin and lidocaine for neuropathic pain relief. Non-pharmacological strategies include structured exercise and lifestyle modification, neuromodulation approaches such as spinal cord stimulation and transcutaneous electrical nerve stimulation, and multidisciplinary foot-care programs to reduce ulceration and amputation risk. Although these approaches can alleviate symptoms and improve quality of life in some patients, none has demonstrated consistent improvement in objective measures of nerve integrity, highlighting the continued need for disease-modifying therapies. Abbreviations: DPN, diabetic peripheral neuropathy; SNRI, serotonin–noradrenaline reuptake inhibitor; TCA, tricyclic antidepressant; TRPV1, transient receptor potential vanilloid 1; SCS, spinal cord stimulation; TENS, transcutaneous electrical nerve stimulation; NCV, nerve conduction velocity; IENFD, intraepidermal nerve fiber density; NSAIDs, non-steroidal anti-inflammatory drugs; AE, adverse effect; NNT, number needed to treat.

Clinical care is therefore multimodal and symptom-focused, combining pharmacological agents with supportive non-pharmacological interventions such as lifestyle modification, neuromodulation, and structured foot-care programs. While these approaches can improve pain scores, mobility, and quality of life in selected patients, average treatment effects remain modest, and interindividual response variability is high, with fewer than half of patients achieving sustained clinically meaningful pain relief across drug classes [87]. Long-term management is further complicated by adverse effects, polypharmacy, renal and cardiovascular comorbidities, and limited adherence, particularly in older populations [43]. Importantly, none of the currently recommended therapies have demonstrated consistent effects on objective markers of nerve integrity, such as nerve conduction velocity or intraepidermal nerve fiber density [18]. The following sections will review current pharmacological and non-pharmacological treatment strategies for DPN (see Figure 2).

### 4.1. Pharmacological Treatment

#### 4.1.1. Analgesics

Conventional non-opioid analgesics, including paracetamol and nonsteroidal anti-inflammatory drugs, are occasionally used in patients with mild symptoms or mixed pain phenotypes but demonstrate minimal efficacy in established painful diabetic peripheral neuropathy [43]. Neuropathic pain is driven primarily by neuronal hyperexcitability, ectopic discharges, and central sensitization rather than peripheral inflammation, rendering cyclooxygenase inhibition largely ineffective [88]. Accordingly, oral nonsteroidal anti-inflammatory Drugs (NSAIDs) lack reliable evidence of benefit for neuropathic pain, with very low-quality data showing no significant reduction in pain intensity. These agents are therefore not recommended as first-line therapy for painful diabetic peripheral neuropathy and, if used, should only be used in short-term treatment [89].

Opioid and opioid-like agents have been studied in painful diabetic peripheral neuropathy but are not recommended for routine use due to limited efficacy and substantial safety concerns [90]. Evidence from randomized trials suggests that short-term opioid treatment may provide modest pain relief in selected patients, as illustrated by a double-blind study of tramadol in which six weeks of therapy at a mean dose of approximately 210 mg per day resulted in greater pain reduction than placebo. However, adverse effects, including nausea, dizziness, constipation, and somnolence, were common [91]. Systematic reviews further indicate that the overall quality of evidence supporting opioids for neuropathic pain is low, benefits are small and short-lived, and there is no evidence of disease modification [91]. Given the well-documented risks of tolerance, dependence, cognitive impairment, falls, endocrine dysfunction, and drug–drug interactions, particularly in patients with diabetes, current clinical guidelines discourage chronic opioid therapy. Opioids should therefore be reserved for short-term use or rescue treatment in carefully selected refractory cases, with close monitoring and predefined discontinuation criteria [90,91].

#### 4.1.2. Anticonvulsants

Gabapentinoids, including gabapentin and pregabalin, are recommended as first-line pharmacological agents for painful diabetic peripheral neuropathy in contemporary guidelines. The analgesic effects are mediated through binding to the α2δ subunit of voltage-gated calcium channels, leading to suppressed excitatory neurotransmitter release [92]. Pregabalin, administered at doses of 300–600 mg per day, produces clinically meaningful pain relief in a subset of patients with painful diabetic peripheral neuropathy, with approximately 30–40% achieving at least a 50% reduction in pain compared with 24–28% with placebo. These outcomes correspond to numbers needed to treat that range from approximately 8 to over 20, depending on dose and outcome definition, underscoring substantial interindividual variability in treatment response. Gabapentin, typically prescribed at a daily dose of 1800–3600 mg, demonstrates comparable average efficacy but with greater variability in clinical response, partly attributable to nonlinear absorption and variable bioavailability [93]. However, clinical response is heterogeneous and frequently limited by adverse effects, including dizziness and somnolence in 20–30% of patients. peripheral edema in 8–15%; and dose-dependent weight gain, with cognitive impairment and fall risk posing concerns in older adults, and dose adjustment required in renal impairment [92]. Importantly, longitudinal and guideline-level evaluations consistently show no improvement in nerve conduction velocity, intraepidermal nerve fiber density, or other objective markers of nerve integrity, confirming that gabapentinoids provide symptomatic pain relief without disease modification and highlighting the need for mechanism-targeted therapies [93].

#### 4.1.3. Antidepressants

Antidepressant agents, particularly serotonin-noradrenaline reuptake inhibitors and tricyclic antidepressants, are widely recommended as first-line therapies for painful DPN. Their analgesic effects are thought to be mediated predominantly by inhibiting serotonin and noradrenaline reuptake, which enhances descending inhibitory pain pathways within the central nervous system [94,95]. Duloxetine, one of the few antidepressants formally approved for painful DPN in multiple regulatory jurisdictions, produces clinically meaningful pain relief, with approximately 40–50% of patients achieving at least a 50% reduction in pain intensity at a daily dose of 60 mg, compared with about 20–25% of patients receiving placebo [50,51]. Venlafaxine extended release demonstrates comparable analgesic efficacy at higher noradrenergic doses, 150–225 mg/day, but its use for this indication generally remains off-label [94,96]. Tricyclic antidepressants such as amitriptyline and nortriptyline may offer analgesic efficacy comparable to the first-line agents for painful DPN in some patients, although the supporting evidence is limited and heterogeneous [97,98]. Their clinical utility is constrained by a narrow therapeutic index and frequent dose-limiting adverse effects, including anticholinergic symptoms, sedation, orthostatic hypotension, weight gain, and cardiac conduction abnormalities, particularly in older adults and those with cardiovascular disease [94,98,99,100,101,102]. Drug–drug interactions further complicate long-term use in patients with diabetes who often require multiple medications. As with other pharmacological options for painful DPN, antidepressants provide symptomatic relief without influencing nerve degeneration or disease progression [99].

#### 4.1.4. Topical and Channel-Targeting Therapies

Topical therapies provide localized pain relief with minimal systemic exposure and are particularly useful for patients who cannot tolerate oral agents or who have focal neuropathic pain [103]. High-concentration capsaicin patches (8%) induce reversible de-functionalization of epidermal nociceptive fibers through sustained TRPV1 activation, with randomized trials showing approximately 30–50% pain reduction lasting 8–12 weeks after a single 30–60 min application. At the same time, adverse effects are largely transient and localized [103,104,105]. Lidocaine 5% plasters inhibit voltage-gated sodium channels and suppress ectopic nerve firing, offering modest benefit in selected patients with superficial, well-demarcated pain and a favorable safety profile that supports adjunctive use despite more limited evidence in DPN [106,107,108]. However, the effects of topical therapies are confined to superficial fibers, require repeated application, and do not modify disease progression [109,110]. In parallel, strategies targeting nociceptor-specific sodium ion-channels such as Nav1.7, Nav1.8, and Nav1.9 have shown a mechanistic preclinical rationale [111,112,113], but several Nav1.7-selective agents have not translated into meaningful clinical benefit in DPN, likely due to channel redundancy, compensatory mechanisms, and substantial interindividual variability in pain biology [69]; early data for Nav-1.8 inhibition in diabetic neuropathy are more encouraging, highlighting the need for improved patient stratification and rational combination approaches that address both peripheral and central pain drivers [109,114,115].

### 4.2. Non-Pharmacological Interventions

Non-pharmacological interventions serve as important adjuncts in the management of DPN, acting primarily through risk-factor modification, functional preservation, and prevention of secondary complications rather than providing robust analgesia [116,117]. Structured exercise and lifestyle interventions that improve glycemic control, reduce obesity, and optimize cardiometabolic risk factors can modestly reduce neuropathic symptoms and enhance functional outcomes, with some evidence suggesting improvements in balance, mobility, and selected measures of nerve structure and function, including intraepidermal nerve fiber density, although results across studies remain heterogeneous and DPN has rarely been the primary endpoint [116,117,118,119,120]. In patients with refractory painful DPN, neuromodulation approaches such as transcutaneous electrical nerve stimulation and spinal cord stimulation modulate nociceptive signaling rather than underlying disease mechanisms [116,121], with the strongest evidence for high-frequency (10-KHz), where randomized trials have shown a majority of carefully selected patients achieve at least 50% pain reduction over 6–12 months [122,123]; however, this is balanced against cost, invasiveness, device-related risks, and the requirement for specialized expertise [116,124,125]. Multidisciplinary foot-care programs remain a cornerstone of DPN management in patients with sensory loss, with structured surveillance, patient education, and pressure offloading consistently associated with substantial reduction in foot ulceration and lower-limb amputation rates [126,127,128,129]. In addition, medical nutrition therapy and specific dietary patterns, including Mediterranean-style diets and diets enriched with omega-3 fatty acids, B vitamins, and antioxidant-rich foods, are increasingly recognized as integral components of comprehensive diabetes care. These approaches may beneficially influence metabolic and inflammatory pathways relevant to DPN progression, although randomized trials with neuropathy-specific endpoints remain limited [44,117,130]. Collectively, these non-pharmacological strategies enhance safety, mobility, and quality of life in people with DPN but, in line with current evidence, do not appear to modify the underlying neurodegenerative process once established [116,117,126].

### 4.3. Modern Antidiabetic Agents and Neuropathic Implications

GLP-1 receptor agonists (GLP-1RAs) have garnered the most cohesive set of novel research regarding their mechanisms. Dhanapalaratnam et al. conducted a prospective human neurophysiology study demonstrating that a 3-month treatment with semaglutide or dulaglutide significantly improved the Total Neuropathy Score (TNS: 3.7 → 2.3, *p* = 0.005) and sural nerve amplitude (11.9 → 14.2 µV, *p* = 0.013). Axonal excitability modeling specifically indicated enhanced Na^+^/K^+^-ATPase pump function and reduced Na^+^ permeability as mechanistic correlates, independent of glycemic improvement [131]. The direct neurophysiological evidence is substantiated at the meta-analytic level by Fan et al., whose systematic review and meta-analysis of six randomized controlled trials (n = 271) revealed that GLP-1RA treatment enhanced nerve conduction velocity by an average of 1.74 m/s (95% CI: 1.16–2.33, *p* < 0.001) relative to controls, with no simultaneous alteration in blood glucose, thereby affirming a glucose-independent neuroprotective mechanism [132]. In preclinical studies, semaglutide mitigated diabetic neuropathic pain in streptozotocin-induced rats by inhibiting spinal cord microglial and astrocytic activation, thus diminishing neuroinflammation-induced allodynia [133]. Liraglutide independently facilitated axonal regeneration and functional recovery after peripheral nerve crush injury in rats via anti-apoptotic regulation of GDF-11 signaling [134]. A complementary preclinical study further established that both semaglutide and dapagliflozin enhanced nerve conduction velocity and decreased nerve tissue steatosis in obese rats through the activation of nerve growth factor (NGF)/synaptophysin and the Nrf2/HO-1 antioxidant pathway [135]. The recent discovery incorporates SGLT2 inhibitors into the preclinical neuropathy context; a recent brief clinical trial also demonstrated that SGLT2 inhibition markedly decreased hydroxyl radical markers and enhanced current perception threshold (CPT) in patients with established neuropathy, indicating a reduction in oxidative nerve damage [136]. Nonetheless, there is no pre-specified, sufficiently powered randomized controlled trial for the SGLT2 inhibitor class concerning neuropathy, and the post hoc analysis of CREDENCE revealed no substantial effect regarding neuropathy endpoints [137]. A comprehensive narrative review by Panou et al. elucidates the incretin domain, indicating that GLP-1 receptor agonists and DPP-4 inhibitors enhance intraepidermal nerve fiber density (IENFD) and neurite outgrowth in experimental models, while recognizing the ongoing necessity for extensive clinical validation [138]. The obesity–inflammation axis persists as an autonomous neuropathic catalyst: Piccolo et al. identified saturated fatty acid lipotoxicity within metabolic syndrome as a direct peripheral nerve stressor [139], Wu et al. confirmed visceral fat area as an independent risk factor for diabetic peripheral neuropathy in type 2 diabetes mellitus [140], and Feldman et al. demonstrated that dietary saturated fatty acid composition alters the gut microbiome in manners that independently correlate with peripheral nerve injury in obese mice, thereby broadening the mechanistic framework beyond traditional glucocentric paradigms [141]. Mendelian randomization further substantiates causal associations among obesity, dyslipidemia, and diabetic neuropathy [142], together emphasizing the imperative of addressing metabolic risk completely in conjunction with pharmaceutical intervention.

## 5. Emerging Disease-Modifying Therapies

**Figure 3:**
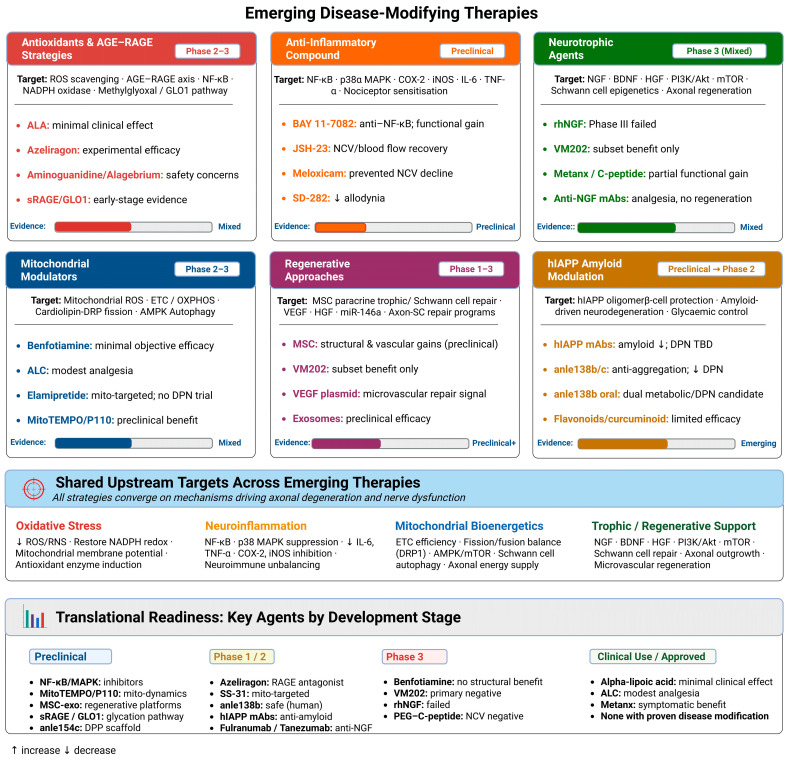
Emerging disease-modifying therapeutic strategies for diabetic peripheral neuropathy (DPN). The schematic summarizes emerging therapeutic strategies targeting DPN, organized by mechanistic class and developmental stage. These include antioxidants and AGE–RAGE pathway modulators; anti-inflammatory agents targeting NF-κB/MAPK signaling (e.g., COX-2, iNOS, IL-6, and TNF-α pathways); neurotrophic therapies involving NGF, BDNF, HGF, PI3K/Akt, and mTOR signaling; mitochondrial modulators affecting ETC/OXPHOS bioenergetics, DRP1-mediated dynamics, and AMPK pathways; regenerative approaches including mesenchymal stem cell (MSC)-based and VEGF- or miR-146a–associated strategies; and inhibitors of human islet amyloid polypeptide (hIAPP) aggregation. Although several candidates demonstrate preclinical or early clinical signals on neuropathic symptoms, motor and sensory nerve conduction (MNCV, SNCV), nerve conduction velocity (NCV), vibration perception threshold (VPT), or intention-to-treat (ITT) outcomes, most remain in early translational phases, and none has yet established consistent disease-modifying efficacy in large-scale trials, including in type 2 diabetes mellitus (T2DM). Abbreviations: ALA, alpha-lipoic acid; AGE, advanced glycation end-products; AMPK, AMP-activated protein kinase; BDNF, brain-derived neurotrophic factor; COX-2, cyclooxygenase-2; DPN, diabetic peripheral neuropathy; DRP1, dynamin-related protein 1; ETC, electron transport chain; GLO1, glyoxalase-1; HGF, hepatocyte growth factor; hIAPP, human islet amyloid polypeptide; IL-6, interleukin-6; iNOS, inducible nitric oxide synthase; ITT, intention-to-treat; MAPK, mitogen-activated protein kinase; MNCV, motor nerve conduction velocity; MSC, mesenchymal stem cell; mTOR, mechanistic target of rapamycin; NCV, nerve conduction velocity; NF-κB, nuclear factor kappa B; NGF, nerve growth factor; OXPHOS, oxidative phosphorylation; PI3K/Akt, phosphoinositide 3-kinase/Akt pathway; RAGE, receptor for advanced glycation end-products; rhNGF, recombinant human nerve growth factor; ROS, reactive oxygen species; sRAGE, soluble receptor for advanced glycation end-products; SNCV, sensory nerve conduction velocity; T2DM, type 2 diabetes mellitus; TNF-α, tumor necrosis factor-alpha; VPT, vibration perception threshold; VEGF, vascular endothelial growth factor; miR-146a, microRNA-146a.

Recent research has identified potential therapies that target the pathogenic mechanisms of DPN. Antioxidant and anti-inflammatory compounds aim to reduce oxidative stress and neuroinflammation, whereas mitochondrial modulators and neurotrophic agents are intended to support neuronal function, bioenergetic, and repair [29,143]. Advanced regenerative strategies, including stem cell therapies and gene-based approaches, remain promising but largely investigational approaches for repairing damaged nerves and modifying disease progression [29,143]. To evaluate the translational maturity of disease-modifying candidates in DPN, a structured evidence hierarchy is necessary. In this section, therapies are grouped into four tiers: Tier I includes agents supported only by mechanistic or in vitro data; Tier II includes agents with efficacy signals in preclinical animal models; Tier III includes agents with early human evidence, such as open-label studies, Phase I/II trials, small randomized controlled trials (RCTs), or post hoc analyses that are insufficient to establish clinical proof of concept; and Tier IV includes agents tested in adequately powered, placebo-controlled RCTs. These tiers are intended to distinguish mechanistic plausibility from clinical readiness. Tier I and Tier II findings should be interpreted as hypothesis-generating or preclinical unless supported by DPN-specific human data. Tier III findings indicate early clinical signals but not definitive proof of disease modification. Tier IV evidence indicates testing in adequately powered placebo-controlled trials; however, even Tier IV classification does not imply efficacy when trial outcomes are negative or inconclusive. The prevailing conclusion from the existing literature is that no single agent has attained consistent, replicated Tier IV evidence for structural nerve protection in diabetic peripheral neuropathy (DPN); most candidates examined below remain within Tiers I–III, whereas a limited number of Tier IV trials have produced negative or inconclusive outcomes. These therapeutic strategies will be discussed in the following sections (Figure 3) (see Table 2).

### 5.1. Antioxidants and AGE–RAGE-Targeted Strategies

Oxidative stress is a central downstream mediator of multiple hyperglycemia- and dyslipidemia-driven pathways, making antioxidant therapy an attractive disease-modifying approach in DPN [144]. Small molecules that scavenge reactive oxygen and nitrogen species, enhance endogenous antioxidant defenses, or improve mitochondrial redox balance have shown neuroprotective effects in experimental models, but translating these effects into consistent clinical benefit has been challenging [145]. A promising mechanistic way is the advanced glycation end-product (AGE)–RAGE axis, which contributes to oxidative stress, neuroinflammation, and microvascular dysfunction in diabetic neuropathy models [29]. Early AGE-directed agents, including dicarbonyl scavengers such as aminoguanidine and cross-link breaker approaches such as alagebrium, improved vascular and neural endpoints preclinically (Tier I), but clinical development has been constrained by safety issues and uncertain long-term efficacy [146]. More recently, small-molecule RAGE antagonists such as azeliragon (TTP488) have shown antinociceptive and neuropathy-relevant benefits in experimental diabetic neuropathy, supporting RAGE blockade as a plausible disease-modifying strategy; however, clinical translation remains unproven, and phase 3 testing in Alzheimer’s disease was halted for lack of efficacy, highlighting the need for DPN-focused trials with biomarker-linked target engagement and objective nerve outcomes [147,148]. Meaning azeliragon cannot be classified above Tier II for DPN and DPN-focused trials with biomarker-linked target engagement, and objective nerve outcomes remain needed. In parallel, non-antagonist approaches are emerging, including soluble RAGE decoys (sRAGE or engineered RAGE ectodomain constructs) that sequester AGEs and other RAGE ligands to blunt downstream signaling, which remain preclinical in the context of DPN, and upstream dicarbonyl-stress modulation targeting methylglyoxal burden and glyoxalase-1 (GLO1) pathways, where early human studies demonstrate target engagement on glycation-related biomarkers [149,150]. Overall, AGE- and RAGE-directed strategies remain mechanistically appealing, but most programs are still preclinical or in early translational stages, and previous setbacks highlight the need for biomarker-linked trials with objective neuropathy measure endpoints.

### 5.2. Anti-Inflammatory Compounds

Anti-inflammatory strategies in DPN are being pursued because hyperglycemia and oxidative stress activate neuroimmune hubs, such as NFκB and MAPK, amplifying cytokines, including IL-6 and TNFα, and promoting nociceptor sensitization and nerve dysfunction [45]. In streptozotocin-induced diabetic neuropathy models, pharmacologic NFκB inhibition has produced functional and biochemical improvements: BAY 11 7082 given orally at 1 or 3 mg/kg/day for 2 weeks ameliorated abnormal sensory responses and nerve functional deficits while reducing sciatic nerve NFκB pathway activation and lowering IL 6, TNFα, COX 2, and iNOS levels, and JSH 23 (1 or 3 mg/kg/day, 2 weeks, oral) reversed nerve conduction and nerve blood flow deficits while similarly reducing elevated IL 6, TNFα, COX 2, and iNOS [57]. Targeting downstream inflammatory enzymes and stress kinases has also shown measurable readouts: selective COX 2 inhibition with meloxicam (1 mg/kg/day, 4 weeks, oral) prevented diabetes-induced motor nerve conduction slowing and endoneurial blood flow deficits, while p38α MAPK inhibition with SD 282 (15 or 45 mg/kg/day, 1 week, i.p.) corrected mechanical allodynia in STZ diabetic rats [151]. Together, these preclinical data indicate that NFκB and p38 MAPK suppression, through cytokine reprogramming, can improve pain behaviors and neurovascular or electrophysiologic surrogates in diabetic models and also underline the need for DPN-focused translation using objective nerve endpoints and durable disease-modifying readouts [45]. Collectively, all agents examined in this subsection currently occupy Tier I–II; no compound has undergone assessment in a powered Phase III RCT with predetermined DPN endpoints, and the existing evidence is solely derived from preclinical rodent models.

### 5.3. Neurotrophic Agents

Neurotrophic insufficiency is increasingly recognized as a contributing mechanism in DPN, in which chronic hyperglycemia and metabolic stress reduce neurotrophin availability, impair retrograde axonal transport, and disrupt downstream survival pathways, such as PI3K/Akt and mTOR signaling [29]. Decreased expression of NGF and BDNF, along with epigenetic modulation of trophic signaling in Schwann cells, contributes to neuronal atrophy, impaired axonal regeneration, and limited repair capacity [152]. Augmentation of neurotrophin pathways has therefore been explored as a disease-modifying strategy. In preclinical models, exogenous NGF or BDNF enhances neurite outgrowth, improves nerve conduction velocity, and promotes axonal regeneration after injury [153]. However, clinical translation of neurotrophin-based therapies has proven challenging. A large randomized, double-blind phase III trial of recombinant human NGF involving 1019 patients with DPN failed to demonstrate significant improvement in the primary neuropathy impairment endpoint despite encouraging preclinical data, highlighting limitations related to systemic delivery and pharmacokinetics [154]. Similarly, the phase III VM202 program (donaperminogene seltoplasmid), a plasmid encoding hepatocyte growth factor, produced mixed results: in the main 9-month trial (intention-to-treat n = 500; 336 VM202, 164 placebo), the primary pain endpoint was not met, whereas a preplanned extended follow-up subset (n = 101) demonstrated statistically significant and clinically meaningful pain reduction with durable benefit after the final injection, highlighting both the biological potential and translational complexity of trophic gene therapy approaches in DPN [155]. Notably, modulation of NGF signaling has also intersected with pain biology; anti-NGF monoclonal antibodies such as fulranumab and tanezumab demonstrated analgesic signals in phase 2 trials in painful DPN (Tier III), though without evidence of structural nerve restoration [156].

More recent strategies aim to enhance endogenous trophic programs or improve tissue-targeted delivery [152]. Metabolic cofactor combinations such as Metanx have been associated with improvements in neuropathic symptoms and quality-of-life metrics in preclinical diabetic models (Tier II) [157]. Long-acting PEGylated C-peptide reduced vibration perception thresholds over 12 months in type 1 diabetes, although primary nerve conduction endpoints were not met [29,158]. These translational experiences underline both the biological rationale and the complexity of targeting neurotrophic pathways in DPN, reinforcing the need for sustained, tissue-specific delivery systems capable of restoring trophic signaling while minimizing systemic exposure [159]. Thus, neurotrophic therapies demonstrate a recurring translational pattern in DPN, specifically, a solid biological rationale paired with mixed or limited late-stage clinical efficacy.

### 5.4. Mitochondrial Modulators

Mitochondrial dysfunction and impaired bioenergetics are increasingly recognized as central features of DPN, linking chronic metabolic stress to excess mitochondrial ROS, impaired oxidative phosphorylation, and activation of downstream stress programs that converge on axonal degeneration and Schwann cell injury, particularly in long peripheral nerves with high energetic demand [160]. The mitochondrial modulators can be organized into redox-directed therapies, metabolic and substrate support strategies that reduce hyperglycemia-linked metabolic overload, and interventions aimed at restoring mitochondrial quality control and stress handling [143]. Clinically, alpha-lipoic acid (typically 600 mg/day) remains the most extensively studied redox-directed agent; however, a recent Cochrane review (3 trials, 816 participants) concluded that ALA probably has little or no effect on neuropathy symptoms at 6 months (Tier IV, negative), emphasizing the gap between oxidative stress targeting and durable clinical benefit [161]. Benfotiamine has been evaluated as a metabolic support approach, with short-term randomized data showing dose-dependent symptom signals. In BENDIP, 165 patients with distal symmetric DPN were randomized to benfotiamine 600 mg/day, 300 mg/day, or placebo for 6 weeks; the primary Neuropathy Symptom Score differed between groups in the per-protocol analysis, was borderline in the intention-to-treat analysis, and there was no clear separation on secondary symptom measures or objective nerve outcomes over this short interval [162]. Longer-term data have been mixed on objective nerve outcomes: in the 12-month double-blind BOND trial (benfotiamine 300 mg twice daily vs. placebo), 57 participants were randomized, and the primary morphometric endpoint (corneal nerve fiber length by corneal confocal microscopy) did not differ between groups, with similarly null effects across multiple secondary morphometric, neurophysiological, and clinical measures (only a trend for symptom score improvement vs. placebo), placing benfotiamine at Tier IV with a negative structural endpoint results [163].

Additional candidates include acetyl-L-carnitine, supported by randomized trials and meta-analyses indicating modest pain reduction with variable evidence for longer-term benefit and heterogeneous study designs (Tier III) [164]. Beyond systemic redox and metabolic approaches, preclinical work implicates disrupted mitochondrial quality control in Schwann cells, involving altered autophagy programs and AMPK/mTOR-related pathways, as actionable regulators; for example, docosahexaenoic acid modulates AMPK-dependent signaling and autophagy responses in Schwann cell models under oxidative stress, though this remains a Tier II observation in the DPN [165]. Mitochondria-focused interventions remain mechanistically compelling in DPN, but mixed clinical outcomes support prioritizing better endpoint selection, improved trial design, and biomarker-linked stratification and, where feasible, more nerve-targeted delivery strategies [143].

Recent efforts have begun to move beyond systemic antioxidants and metabolic supplements toward mitochondria-directed pharmacology that either accumulates within mitochondria or directly targets inner-membrane architecture and fission machinery. Elamipretide (SS-31) is a cardiolipin-binding peptide that localizes to the inner mitochondrial membrane and is intended to stabilize cristae structure and improve respiratory efficiency; it is in clinical-stage trials for primary mitochondrial diseases but has not yet been established in dedicated DPN clinical trials (Tier II) [166]. MitoTEMPO is a triphenylphosphonium-linked nitroxide that concentrates in mitochondria to quench mitochondrial ROS, and recent preclinical studies in diabetic neuropathy models report improvements in nerve structure and electrophysiologic readouts, so it should be framed as preclinical for DPN (Tier II) [167]. Targeting mitochondrial dynamics, the DRP1-FIS1 interaction inhibitor peptide P110 is a selective fission-modulating approach with expanding preclinical evidence across injury and neurodegeneration models and recent mechanistic literature. Still, DPN-specific validation remains limited, so it remains preclinical in the DPN context [168]. Finally, while mdivi-1 is widely cited as a “fission inhibitor,” recent mechanistic work indicates it acts largely as a reversible complex I inhibitor with off-target bioenergetic effects rather than a clean DRP1 inhibitor, so it is best presented as a tool compound that supports target rationale but is not a mature therapeutic lead for DPN [168]. Mitochondrial modulators remain promising, with the strongest clinical evidence supporting symptom-focused metabolic agents and much weaker support for mitochondria-targeted compounds, most of which are in preclinical stages.

### 5.5. Regenerative Approaches (Stem Cell Therapies and Gene-Based Approaches)

Regenerative approaches in DPN aim to restore lost trophic support, improve microvascular supply, and reactivate axon-Schwann cell repair programs rather than only suppress symptoms [169]. Across recent reviews, stem cell therapy is positioned as a predominantly paracrine strategy: MSCs are highlighted for immunomodulatory and neurotrophic secretomes, with consistent preclinical signals of improved nerve function and pain-related outcomes in diabetic models [170]. There are improvements in electrophysiological and sensory readouts, including nerve conduction parameters and vibration perception threshold, although limited blinding and other potential sources of bias constrain causal inference [171]. From the meta-analysis of preclinical studies, MSC treatment was associated with clear improvements in diabetic neuropathy outcomes, including motor and sensory nerve conduction velocities, intraepidermal nerve fiber density, sciatic nerve blood flow, and the capillary-to-muscle fiber ratio (Tier II–III) [171].

On the other hand, gene-based regenerative approaches have advanced further in human clinical trials, primarily through local delivery of pro-regenerative growth factors. VM202 (an HGF-encoding plasmid) was well tolerated but did not meet efficacy endpoints in the main phase 3 study. At the same time, a smaller extended follow-up cohort showed a statistically significant reduction in pain compared with placebo, highlighting persistent gaps between analgesic benefit and broader disease-modifying endpoints [155,172]. Complementary vascular-regenerative strategies are best framed using DPN-specific trials, such as plasmid VEGF approaches evaluated in diabetic neuropathy [173]. Finally, cell-free regenerative biologics are increasingly emphasized in preclinical DPN, including engineered MSC-derived exosomes enriched with miR-146a and Schwann cell-derived exosome platforms, which converge on inflammatory and pro-survival signaling to improve neuropathy phenotypes in diabetic models but still require translational optimization of dosing, potency assays, and manufacturing consistency (Tier I–II) [174]. Evidence grading across regenerative modalities remains uneven. Most stem cell, iPSC-derived products, and exosome or miRNA-based approaches are still preclinical, with efficacy supported mainly by diabetic rodent models and heterogeneous, non-standardized endpoints, so they should be presented as mechanistically promising but not clinically validated [175]. Therefore, regenerative therapies should now be viewed as promising but not yet clinically proven disease-modifying approaches for DPN.

### 5.6. Targeting Human Islet Amyloid Polypeptide (hIAPP)

One emerging hypothesis-generating axis focuses on hIAPP, a biologically plausible contributor to both metabolic dysregulation and neuropathic injury. hIAPP aggregation leads to β-cell dysfunction and systemic metabolic toxicity, which may indirectly exacerbate peripheral nerve damage [7]. While small organic molecules and natural products such as flavonoids and curcuminoids have demonstrated anti-aggregation activity against hIAPP in vitro, their limited efficacy and absence of demonstrated β-cell protection restrict their translational potential. Similarly, peptide-based inhibitors, including pramlintide and modified IAPP analogues, have shown the ability to interfere with early amyloid formation but have been hampered by modest effectiveness and adverse effects in preclinical settings. More compelling preclinical results have been obtained with hIAPP-specific monoclonal antibodies that selectively target toxic oligomeric or protofibrillar species [7]; for example, in hIAPP transgenic mouse models, such antibodies reduce amyloid burden, protect pancreatic β-cells, improve glucose homeostasis, and prolong survival. Although their direct effects on DPN endpoints, such as intraepidermal nerve fiber density or nerve conduction, remain to be established in dedicated studies, early elimination of soluble hIAPP aggregates may represent a disease-modifying approach by addressing upstream drivers of both metabolic and neural dysfunction. In parallel, small-molecule amyloid modulators derived from di-phenyl-pyrazole (DPP) scaffolds, including anle138b and anle154c, inhibit hIAPP aggregation by modulating β-sheet formation and oligomer stability. In preclinical models, these agents not only prevent hIAPP-induced fibril formation and mitochondrial dysfunction but also attenuate neuropathic phenotypes, including mechanical allodynia and intraepidermal nerve fiber loss, and oral anle138b has demonstrated improvements in glycemic control and pancreatic islet function alongside a favorable human safety profile in non-DPN contexts, positioning DPP compounds as investigational disease-modifying candidates warranting DPN-focused translational studies [7].

Rigorous clinical evaluation, including DPN-specific outcome measures and biomarker strategies to confirm target engagement in nerve tissue, will be required before hIAPP-directed therapies can be positioned beyond Tier I in this indication.

**Table 2:** Summary of emerging disease-modifying therapeutic strategies for diabetic peripheral neuropathy: targets, mechanisms, evidence tier, and key limitations.

Therapeutic Class	Representative Agents	Primary Target/Mechanism	Highest Evidence Stage	Key Limitation(s)
Antioxidants/AGE–RAGE Inhibitors	Alpha-lipoic acid (ALA); Benfotiamine; Azeliragon (TTP488)	Reduction in oxidative stress; inhibition of AGE formation and RAGE-mediated signaling	Tier IV (RCT)	Heterogeneous trial results; no regulatory approval as a disease-modifying agent; effect sizes modest; long-term efficacy unconfirmed
Anti-inflammatory Agents	BAY 11-7082 (NF-κB inhibitor); Meloxicam (COX-2 inhibitor); p38 MAPK inhibitors	Suppression of NF-κB, COX-2, TNF-α, IL-1β; modulation of neuroinflammatory cascades	Tier I–II (preclinical)	Limited human data; systemic toxicity concerns non-selective agents; DPN-specific RCTs absent; incomplete target specificity
Neurotrophic Agents	Recombinant NGF; VM202/HGF plasmid; Metanx (L-methylfolate formulation); PEGylated C-peptide	Restoration of neurotrophic support; HGF-mediated axonal survival and angiogenesis; methylation cycle support; insulin-mimetic effects	Tier IV (RCT for NGF); Tier III (VM202, Metanx, C-peptide)	NGF RCTs failed primary endpoints; VM202 requires intramuscular delivery; C-peptide development discontinued; evidence is heterogeneous
Mitochondrial Modulators	Alpha-lipoic acid (ALA); Acetyl-L-carnitine (ALCAR); Elamipretide (SS-31); MitoTEMPO; P110 (DRP1 inhibitor)	Reduction in mitochondrial ROS; restoration of oxidative phosphorylation; cardiolipin stabilization; DRP1-mediated mitochondrial fission inhibition	Tier IV (ALA); Tier II–III (ALCAR); Tier I–II (elamipretide, MitoTEMPO, P110)	ALA evidence mixed across trials; elamipretide, MitoTEMPO, and P110 have no clinical DPN RCTs; lack of validated mitochondrial endpoint; DPN-specific trial data absent for newer agents
Regenerative/Stem Cell Approaches	Mesenchymal stem cells (MSC); iPSC-derived neural progenitors; MSC-derived exosomes	Paracrine neurotrophic and angiogenic support; neural differentiation potential; exosome-mediated miRNA transfer	Tier III (MSC); Tier I–II (iPSC, exosomes)	Small pilot studies only; manufacturing complexity; immunological considerations; route of delivery; no completed DPN RCTs; long-term safety data absent
hIAPP-Targeting Strategies	Anti-hIAPP monoclonal antibodies; Anle138b/DPP small-molecule aggregation inhibitors; Pramlintide (amylin analogue)	Prevention of hIAPP fibril formation; disruption of amyloid aggregation; amylin receptor antagonism	Tier I–II (preclinical only)	No clinical DPN trials; uncertain magnitude of hIAPP contribution to clinical DPN; pramlintide is an amylin analogue agonist—repurposing logic for DPN remains speculative
Modern Antidiabetic Agents	GLP-1 receptor agonists (semaglutide, liraglutide, dulaglutide); SGLT2 inhibitors (canagliflozin, empagliflozin, dapagliflozin)	GLP-1R-mediated neuroprotection, anti-neuroinflammation, Na^+^/K^+^-ATPase restoration; SGLT2i-mediated ketogenesis, AMPK activation, oxidative stress reduction	Tier III–IV (GLP-1RA: REWIND, prospective human neurophysiology, meta-analysis); Tier II–III (SGLT2i: preclinical + CREDENCE post hoc)	No prespecified powered DPN RCT for either class; GLP-1RA evidence strongest but requires replication; SGLT2i CREDENCE post hoc null; combination strategies remain preclinical

### 5.7. Why Disease-Modifying Trials Fail: Endpoint and Design Challenges

Across these therapeutic classes, the evidence base supports biological plausibility for several mechanism-based interventions, but it does not yet establish durable structural nerve protection for most candidates in adequately powered clinical trials. Accordingly, emerging therapies should be interpreted according to their evidence tier, with preclinical findings, early-phase clinical signals, and randomized trial outcomes clearly distinguished.

The history of disease-modifying therapy development for DPN is, with few exceptions, a history of clinical trial failure, and this pattern demands systematic analysis rather than dismissal. The most widely used primary endpoint has historically been nerve conduction velocity (NCV), a measure of large-fiber electrophysiological function that is relatively insensitive to early DPN where injury is confined to small myelinated and unmyelinated fibers and which does not reliably reflect the pain, thermal sensation, and autonomic dysfunction most relevant to patient quality of life [18,30]; the ADA DPN position statement has explicitly acknowledged that NCV deteriorates slowly and non-linearly in established disease, demanding prolonged follow-up periods to detect meaningful change and making it poorly suited as a primary endpoint in trials of finite duration [18]. Intraepidermal nerve fiber density (IENFD), measured by skin punch biopsy, provides a more sensitive direct histological measure of small unmyelinated C-fiber integrity and has demonstrated responsiveness to lifestyle interventions targeting metabolic risk [118]; however, IENFD measurement is limited by significant sampling variability and has not achieved regulatory acceptance as a validated surrogate endpoint, a fundamental infrastructure barrier that constrains its use as a registration-trial primary endpoint [31]. Corneal confocal microscopy (CCM), quantifying sub-basal nerve plexus fiber density and morphology non-invasively, has emerged as the most promising endpoint technology for detecting early structural change and monitoring intervention response, with systematic evidence supporting its sensitivity to DPN progression and its applicability across disease stages; wider regulatory qualification of CCM is a priority for the field [80,81]. Pain numeric rating scales, while regulatory accepted for symptomatic endpoints, are affected by placebo response rates of 30–50% in painful DPN trials and require enriched enrollment and run-in designs to yield interpretable results [89,94,116]. Beyond endpoints, target selection has been systematically flawed: agents including aldose reductase inhibitors [37], PKC-beta inhibitors [40], and antioxidants including alpha-lipoic acid [161] have been evaluated as single-pathway interventions in a condition defined by simultaneous activation of multiple reinforcing biochemical cascades, such that neutralizing one pathway is insufficient to alter the overall trajectory of nerve injury in patients with established multi-hit disease; furthermore, a recent benfotiamine RCT over 12 months found no significant improvement in its primary morphometric endpoint despite a biologically plausible mechanism [163], enforcing the principle that even well-targeted agents may fail when enrollment stage, duration, and endpoint sensitivity are mismatched. Patient heterogeneity compounds these failures: painful versus painless, small-fiber-predominant versus large-fiber-predominant, and early-stage versus late-stage DPN represent mechanistically distinct populations that have been enrolled together without phenotypic stratification, diluting treatment effects in genuinely responsive subgroups [23,27,28]; the metabolic syndrome and obesity constitute independent neuropathic drivers through adipokine dysregulation and lipotoxic nerve injury [176], mechanisms distinct from classical glycaemic pathways and unlikely to respond to glucocentric interventions, yet most trials have not stratified enrollment by adiposity, dyslipidaemia, or inflammatory burden [32,177].

Timing represents an equally critical and underappreciated failure: the majority of DPN RCTs have enrolled patients with established, symptomatic nerve fiber loss, at which point regenerative capacity may be too compromised to demonstrate measurable structural benefit; preclinical evidence consistently demonstrates that neuroprotective interventions, whether targeting mitochondrial fission [168], endoneurial oxidative stress [167], or neurotrophic signaling [178], have time-sensitive biological windows of efficacy that late-enrollment trial designs structurally cannot access, a principle further supported by the observation that GLP-1R agonist-mediated neuroprotection in experimental models depends on intact axonal signaling machinery and adequate Schwann cell viability that decline with disease progression [133,179,180]. Finally, the absence of validated mechanistically linked biomarkers for patient stratification may be the most fundamental deficiency of all: without tools to identify patients whose DPN is driven predominantly by the pathway being targeted, selecting oxidatively stressed patients for antioxidant trials, lipotoxic-obese patients for metabolic intervention trials, or neuroinflammation-dominant patients for anti-inflammatory trials, even pharmacologically effective agents will be diluted across non-responsive populations and generate false-negative outcomes indistinguishable from true pharmacological failure [27,162]. Addressing this stratification gap through the prospective validation of mechanistically linked biomarker panels is the prerequisite without which no advance in endpoint technology or combination therapy design can reach its full translational potential.

## 6. Conclusions

DPN is a multifactorial complication of diabetes, driven by hyperglycemia, metabolic disturbances, vascular insufficiency, and emerging amyloidogenic mechanisms. Although substantial progress has been made in understanding these pathogenic pathways, translation into effective disease-modifying treatments has lagged behind.

Current clinical management is largely centered on symptomatic relief, particularly neuropathic pain control, through pharmacologic and supportive strategies. While these approaches can improve patient-reported outcomes and quality of life in selected individuals, their overall efficacy is modest, interindividual variability is considerable, and none reliably halt or reverse structural nerve damage. As a result, progressive sensory decline and nerve fiber loss often continue despite optimized care.

Encouragingly, emerging therapeutic strategies that directly target upstream metabolic, oxidative, inflammatory, mitochondrial, amyloidogenic, neurotrophic, and regenerative pathways may support a gradual shift from symptom control toward mechanism-based intervention. However, most proposed disease-modifying strategies remain experimental or early translational, and their ability to preserve nerve structure, restore function, or alter long-term disease trajectory remains to be established in adequately powered, phenotype-stratified clinical trials. Continued integration of mechanistic insights with biomarker-enriched patient selection, appropriate endpoints, and well-designed clinical trials will be essential to determine which candidates can progress from experimental concepts to clinically effective therapies capable of improving long-term outcomes in patients with DPN.

## Data Availability

No new data were created or analyzed in this study.
